# Trunk Posture from Randomly Oriented Accelerometers

**DOI:** 10.3390/s22197690

**Published:** 2022-10-10

**Authors:** Aidan R. W. Friederich, Musa L. Audu, Ronald J. Triolo

**Affiliations:** 1 Department of Biomedical Engineering, Case Western Reserve University, Cleveland, OH 44106, USA; 2 Advanced Platform Technology Center, Louis Stokes Veterans Affairs Hospital, Cleveland, OH 44106, USA

**Keywords:** sensor fusion, spinal cord injury, accelerometer, neuroprosthesis

## Abstract

Feedback control of functional neuromuscular stimulation has the potential to improve daily function for individuals with spinal cord injuries (SCIs) by enhancing seated stability. Our fully implanted networked neuroprosthesis (NNP) can provide real-time feedback signals for controlling the trunk through accelerometers embedded in modules distributed throughout the trunk. Typically, inertial sensors are aligned with the relevant body segment. However, NNP implanted modules are placed according to surgical constraints and their precise locations and orientations are generally unknown. We have developed a method for calibrating multiple randomly oriented accelerometers and fusing their signals into a measure of trunk orientation. Six accelerometers were externally attached in random orientations to the trunks of six individuals with SCI. Calibration with an optical motion capture system resulted in RMSE below 5° and correlation coefficients above 0.97. Calibration with a handheld goniometer resulted in RMSE of 7° and correlation coefficients above 0.93. Our method can obtain trunk orientation from a network of sensors without *a priori* knowledge of their relationships to the body anatomical axes. The results of this study will be invaluable in the design of feedback control systems for stabilizing the trunk of individuals with SCI in combination with the NNP implanted technology.

## 1. Introduction

Individuals with paralysis have been able to regain various functions with functional neuromuscular stimulation (FNS), which is the process of applying small electrical currents to peripheral nerves to elicit muscle contraction from the otherwise paralyzed muscles. Through FNS, people with Spinal Cord Injury (SCI) have been given new options to stand and step [1,2], bike [3,4], row [5], maintain upright seated posture [6], and perform reaching activities [7,8]. For review see Karamian et al. [9] or Marquez et al. [10]. Many of these achievements have been accomplished with open loop control where fixed temporal patterns of stimulation parameters are predetermined to realize a specific movement. For example, cycling controllers [11] apply the same set of stimulation pulse amplitudes, durations, and frequencies as a function of crank angle regardless of cadence or number of repetitions. Stereotypical feed-forward controls tend to be blind to internally generated or externally applied disturbances that can destabilize the system. Perturbations, such as trips during walking, bumps while seated, and fatigue while cycling or rowing can degrade performance and user confidence. Incorporating feedback control allows the system to compensate for perturbations by modifying the stimulation commands based on the current system state.

Restoration of trunk stability has been consistently rated highly for those with SCI [12,13,14]. Feedforward control of FNS applied to contract the hip and trunk musculature can expand reaching area [15] and improve wheelchair propulsion efficiency [16]. Yet, seated stability remains an attractive target for feedback control. Incorporating feedback control to maintain seated posture and balance has enabled individuals with SCI to resist both externally applied and internally generated perturbations [17,18,19]. Further advancements for such systems could enable leaning postures that expand the seated workspace and enhance a user’s ability to exert control over objects in the environment otherwise inaccessible from the wheelchair. Incorporating feedback control can provide additional benefits, but requires an accurate measure of the current system state. Inertial measurement units (IMU) are a promising choice for providing this information due to their small size and our ability to accurately infer joint angles from multiple sensors [20]. IMUs are often composed of accelerometers that measure linear accelerations, gyroscopes that measure rotational velocity, and magnetometers to derive heading from the global magnetosphere. All three component signals can then be fused into a measure of sensor orientation [21]. However, these sensor fusion methods are subject to random drift from the integration of the gyroscopic data [22] and ferromagnetic disturbances to the magnetometer readings [23]. Previous integration of IMUs in feedback control of trunk posture has been achieved with an externally-mounted 3-axis accelerometer placed on the chest or the back [18,19,24] to reflect tilt based on the consistent gravity vector compared to the inclined accelerometer coordinate frame. To obtain relevant measures of trunk orientation, the sensor was carefully aligned with the anatomical axes of the body. Such reliance on accurate sensor placement could complicate donning and doffing, thereby making feedback control systems for daily use outside of the laboratory difficult.

Recently, IMUs have been incorporated into a fully implanted neuroprosthesis developed at Case Western Reserve University in Cleveland (Clinical Trial: NCT02329652) [25,26]. Termed the Networked Neuroprosthesis (NNP), it is composed of a network of connected “remote modules” attached to a central “power module” that contains the battery that powers the entire system and the electronic hardware that mediates data processing and radio communication with the outside world. The remote modules can either generate stimulus pulses or record biopotentials through separate electrodes placed near the anatomical targets. Crucially, each remote module also contains a 3-axis accelerometer, allowing the possibility of a fully implanted feedback control system for seated posture without the need to don external sensors. The implanted accelerometers have been shown to be sensitive to trunk tilt [26], and the accuracy of those measurements is currently an ongoing topic of investigation. As an added benefit, the quantitative measures of trunk orientation either from IMUs, motion capture, or other means is valuable for assessing postural control after SCI [27,28,29]. Being able to obtain a reliable measure of postural biomechanics from an implanted system could provide long-term postural information to aid in the rehabilitation and treatment of SCI.

**Figure 1:**
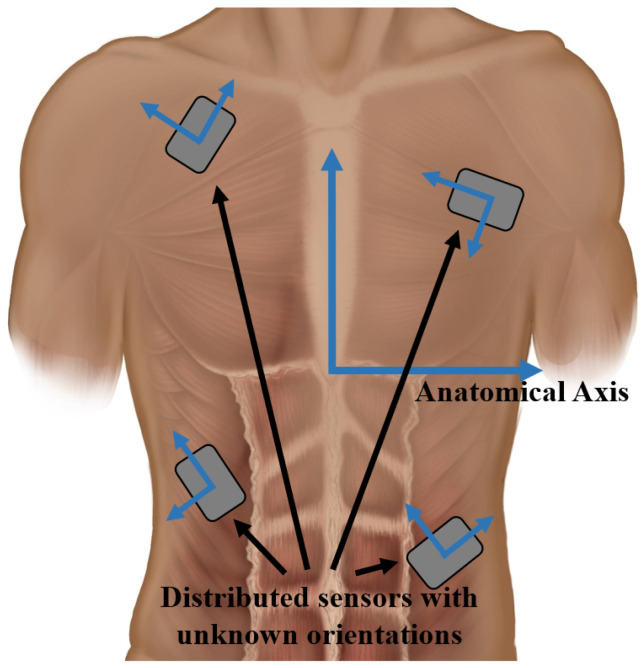
Diagram of a distributed sensor system. The coordinate frames of these sensors are unknown relative to the body’s anatomical coordinate frame.

Obtaining relevant tilt information requires knowledge of the orientation of the accelerometer, typically obtained by aligning the coordinate frames of the sensor and the object of interest. Previous studies of trunk posture have placed the IMUs on the lower lumbar spine [30,31], the sternum [32], and various positions on the back, including at the T10 [33], T4 [34], or T1 vertebra [35]. These studies have all attempted to align the sensor with the body anatomic axes, such that its pitch output refers to trunk extension and flexion and roll refers to trunk lateral bending. However, placement of the NNP remote modules is determined by the surgeon during implantation when they suture the remote modules to the underlying tissue and, thus, the orientation of each sensor is not specifically set to meet any anatomic orientation requirements and is unique to each recipient (Figure 1). For surgeons to precisely align and suture the remote modules in specific orientation would require additional time and complicate an already complex and time-consuming surgery. Moreover, there is no guarantee the remote modules would remain aligned with the anatomy post-surgery as the recipient resumes an upright posture and their daily seated activities. Any movement of modules will be minimized once the individual is fully healed, and the modules are encapsulated in the body tissue. Although obtaining module orientation post-implantation through imaging is possible, it would require multiple images across multiple implant sites and sophisticated image processing to relate them to the anatomy in three dimensions. A simpler method of calibrating and fusing the contributions of multiple potentially inaccurate sensors into one global trunk orientation signal is clearly needed to facilitate eventual clinical deployment of the NNP.

In this study, we have developed and verified a method of calibrating and fusing signals from multiple accelerometers with unknown orientations into an accurate measure of trunk posture. The method first aligns the sensor signals with the body anatomic axes, then converts the signals to sensor pitch and roll, and finally fuses them into a single measure of overall trunk orientation. The process can be completed with either a motion capture system for high fidelity or an inexpensive handheld goniometer common in clinical settings for at home applications.

## 2. Materials and Methods

### 2.1. Participants

Six individuals with SCI participated in this study. We recruited individuals with SCI to closely reflect the targeted population of potential recipients of the NNP for trunk control applications. Able-bodied subjects were not considered because SCI significantly alters both the active and passive mechanics of the paralyzed muscles and, thus, the biomechanics of the trunk [36]. Subject neurological and anthropometric characteristics are presented in Table 1. Participants were informed of all aspects of the experiment and subsequently signed consent forms approved by the local institutional review board (IRB: VA Northeast Ohio Healthcare System, Protocol Number: 1591730, Approval Date: 7 February 2021).

### 2.2. Experiment Setup

**Figure 2:**
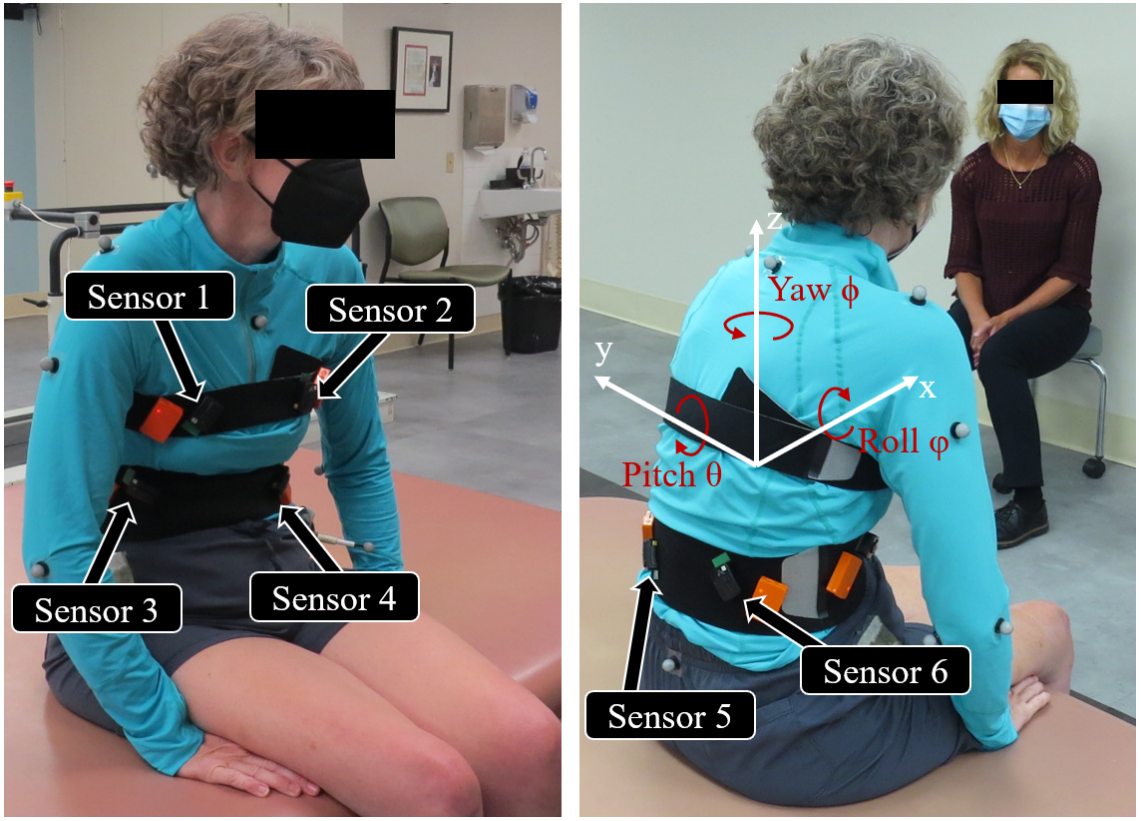
Locations of the 3-axis accelerometers and reflective markers on the subject. The body’s anatomical coordinate frame is shown in the right image. Pitch, roll, and yaw refer to trunk flexion and extension, lateral bending, and axial rotation about the x, y, and z anatomical axes, respectively.

We placed six 3-axis accelerometers (Trigno Avanti, Delsys Inc., Natick, MA, USA) with double sided tape in the general vicinity to where the NNP remote modules would be implanted in the trunk [26] as informed by the first recipients of the prototype system. These included both right and left sides of the chest and the front and rear abdomen. Figure 2 shows the accelerometer placement. Sensors were placed in random unquantified orientations and their precise locations were not recorded beyond their general vicinity. The case dimensions of the Delsys accelerometer casing is 27 × 37 × 15 mm compared to the remote module casing of 55 × 15 × 5 mm [26]. However, within the casings the accelerometers are roughly 3 × 3 mm. In addition, reflective markers were placed on the C7 vertebrae, sacrum, and bilaterally on the acromion of the scapula, greater trochanter of the femur, middle of the upper arm, the lateral and medial epicondyle of the elbow, and the anterior superior iliac spine of the pelvis. Accelerometer data were transmitted wirelessly to the Trigno Base System that relayed these signals through an analog connection to a Speedgoat real-time computer (Performance real-time target machine, Speedgoat, Switzerland). The Speedgoat system sampled the data at 100 Hz with a custom Simulink model (Mathworks, Natick, MA, USA). At the beginning of each trial the Simulink model sent a +3.3 V pulse through the Speedgoat digital output block to the motion capture system to synchronize the signals from the two systems. Trunk kinematics were obtained from a 16-camera motion capture system sampling the marker positions at 100 Hz (Vicon Motion Systems Ltd., Oxford, UK). Once the sensors and markers were placed, the participant sat on an examination table situated in the middle of the work volume of the motion capture system.

### 2.3. Motion Capture Calibration

**Figure 3:**
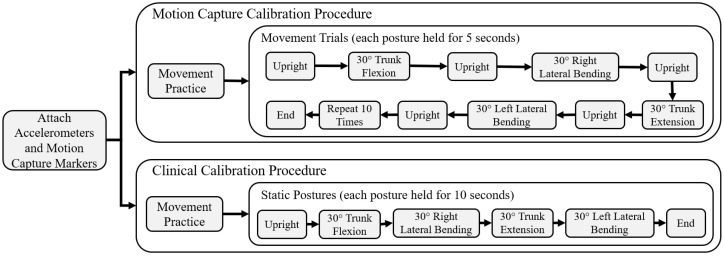
Experimental procedure flowchart for both the motion caption and clinical calibration methods.

Subjects started in an upright posture for five seconds and proceeded to move to 30° in forward trunk flexion, right bending, trunk extension, and left bending for five seconds each. Each change in posture was separated by returning upright for five seconds. Figure 3 contains a flowchart of the experimental procedure. Subjects were allowed to use their arms or the help of an experimenter to support themselves during these movements. A full completion of the sequence of all movements was considered a single trial. The movement sequence was repeated 10 times, with half intended for the calibration process and the other half for testing. An experimenter first demonstrated the movement pattern, and the subjects were allowed to practice as many times as necessary until they felt comfortable and competent in its performance. During practice, a medical goniometer was set at 30° as a reference. During the recorded movements, the 30° target was not strictly enforced as the motion capture system recorded the true angles.

**Figure 4:**
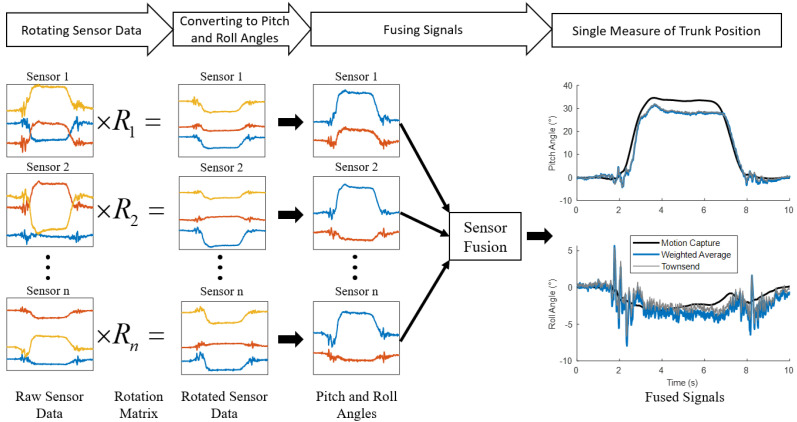
Overview of the sensor rotation and fusion process. Signals from each accelerometer are first rotated, then converted to pitch and roll angles. Finally, the pitch and roll angles from every sensor are fused into a single measure of trunk position.

The computational aspect of the calibration process entailed three main steps shown in Figure 4. These steps were performed separately for each subject, as the sensor orientation was unique to each subject. First, the accelerometer signals were rotated to align the local sensor coordinate frame with the body coordinate frame. Second, the linear accelerations along the three axes of each sensor were converted to sensor pitch and roll angles. Finally, the pitch and roll angles from all six sensors were fused into a single measure of trunk pitch and roll.

Equations (1)–(3) show the conventional rotation matrices which will transform a vector by ϕ, θ, ψ about the axes *x*, *y*, and *z*, respectively.

(1) $$R_{x}\left(\phi \right)=\left[\begin{array}{ccc} 1 & 0 & 0\\ 0 & cos(\phi ) & sin(\phi )\\ 0 & -sin(\phi ) & cos(\phi ) \end{array}\right]$$

(2) $$R_{y}\left(\theta \right)=\left[\begin{array}{ccc} cos(\theta ) & 0 & -sin(\theta )\\ 0 & 1 & 0\\ sin(\theta ) & 0 & cos(\theta ) \end{array}\right]$$

(3) $$R_{z}\left(\psi \right)=\left[\begin{array}{ccc} cos(\psi ) & sin(\psi ) & 0\\ -sin(\psi ) & cos(\psi ) & 0\\ 0 & 0 & 1 \end{array}\right]$$

Multiplying the three rotation matrices creates at rotation matrix (Equation (4)) capable of transforming a vector into any orientation.

(4) $$R\left(\phi ,\theta ,\psi \right)=R_{x}\left(\phi \right)R_{y}\left(\theta \right)R_{z}\left(\psi \right)$$

R(ϕ,θ,ψ) was then multiplied by the raw acceleration signals to obtain a rotated acceleration vector (Equation (5)).

(5) $$\left[\begin{array}{c} a_{rot,x}\\ a_{rot,y}\\ a_{rot,z} \end{array}\right]=R\left(\phi ,\theta ,\psi \right)\left[\begin{array}{c} a_{raw,x}\\ a_{raw,y}\\ a_{raw,z} \end{array}\right]$$

If the correct rotation angles (ϕ,θ,ψ) are chosen the rotated accelerometer coordinate frame will align with the anatomic axes of the body. To determine the correct rotation angles, we first converted the rotated signals to pitch and roll angles corresponding to the orientation of the subject’s trunk with Equations (6) and (7). Optimization was performed jointly on Equations (6) and (7) with a non-linear curve-fitting algorithm in a least-squares sense with the Matlab function lsqcurvefit. Sensor pitch and roll angles were optimized to fit trunk pitch and roll angles obtained from the movement trials marked as training data. These gold-standard trunk pitch and roll angles were determined from motion capture as the angle between the global reference frame and the line defined between the sacrum and C7 marker [37]. The variables optimized were ϕ, θ, and ψ from Equation (4). Lower bounds were set to 0 and upper bounds were set to 2π. As this is a quasi-static analysis, the accelerometer has only two degrees of freedom because we assume the magnitude of the acceleration vector equals 1 g. As a result, the optimization often found local minima. To find a global minimum a MultiStart algorithm was used to generate multiple initial conditions [38]. This process was applied to each sensor separately.

(6) $$Roll=tan^{-1}\left(\frac{a_{rot,y}}{a_{rot,z}}\right)$$

(7) $$Pitch=tan^{-1}\left(\frac{-a_{rot,x}}{\sqrt{\left(a_{root,y}^{2}+a_{rot,z}^{2}\right)}}\right)$$

Once the signals from each accelerometer were aligned with the trunk anatomic axis and converted to pitch and roll angles, the angles from all the six accelerometers were fused with one of two algorithms. The first, shown in Equation (8), was a weighted average function where the angle ($a_{k}$) was multiplied with a weighting parameter ($w_{k}$). The angles from each accelerometer are then summed and divided by the sum of the weights. The second method employed a Townsend algorithm [39], defined by Equation (9), which was originally derived to fuse heart rate signals from multiple sources in a hospital setting. Fused pitch and roll angles were optimized to minimize least-squares error between the trunk pitch and roll angles obtained from the motion capture during the training movements through a second optimization with a non-linear curve-fitting algorithm in a least-squares sense with the Matlab function lsqcurvefit. The variables optimized were the weights (w) in Equation (8) and weights (σ) in Equation (9). Lower bounds were set to 0 and upper bounds were set to 1 for the weights (w) in Equation (8). Lower bounds were set to −1 and upper bounds were set to 1 for the weights (σ) in Equation (9). The second optimization acts both to reduce overall error and as an objective method for choosing the sensors that best reflect the pitch and roll angles.

(8) $$\bar{a}=\frac{\sum_{k=1}^{n}\left(a_{k}\right)\cdot w_{k}}{\sum_{k=1}^{n}w_{k}}$$

(9) $$\bar{a}=\sum_{k=1}^{n}\left(\frac{\prod_{i=1,i\neq k}^{n}\sigma_{i}^{2}}{\sum_{i=1}^{n}\left(\prod_{j=1,j\neq i}^{n}\sigma_{j}^{2}\right)}\cdot a_{k}\right)$$

### 2.4. Clinical Calibration

For the eventual clinical implementation and widespread dissemination of the seated balance controllers we envisage for NNP recipients, it should be possible to perform the calibration and fusion process outside of a laboratory with expensive motion capture capabilities. We, therefore, explored the possibility of utilizing only equipment typically available in a clinical or home setting. Instead of optimizing the calibration constants to match gold-standard motion capture data, we used a conventional commercially available medical goniometer to assess the various trunk postures. Each subject assumed five postures, lasting 10 s each, while data were collected from each accelerometer. The five postures set via the goniometer were: upright sitting, 30° trunk flexion, 30° right bending, 30° trunk extension, and 30° left bending. Each posture was assumed once. Figure 3 contains a flowchart of the experimental procedure.

The training data were set as the first 2.5 s of each posture and remaining time was discarded. Testing data were the same testing trials collected from the motion caption calibration. The first 2.5 s from each posture were collated into a single matrix. Each timepoint was paired with the target angles set by the goniometer. For example, the data from upright sitting would be paired with 0° pitch and 0° roll angles; the data from 30° trunk flexion would be paired with 30° pitch and 0° roll angles. The same least squares optimization described earlier was applied to the data to determine the angles from Equation (4) by minimizing the error between the targeted posture measured with the goniometer and those indicated by the accelerometers. The optimization process was the same as reported in Section 2.3 above. A second optimization was performed with the fusion Equations (8) and (9) to optimize the weights (w, σ) that minimized the error between the targeted posture measured with the goniometer and those indicated by the now fused accelerometers.

### 2.5. Experiment Data Analysis and Statistics

After optimization, both the motion capture and clinical calibration algorithms were verified with the movement trials set aside for testing from the motion capture calibration section. Measures of trunk position were compared to the motion capture of those same movements with root mean squared error (RMSE) and correlation coefficients (r).

## 3. Results

### 3.1. Motion Capture Calibration

**Figure 5:**
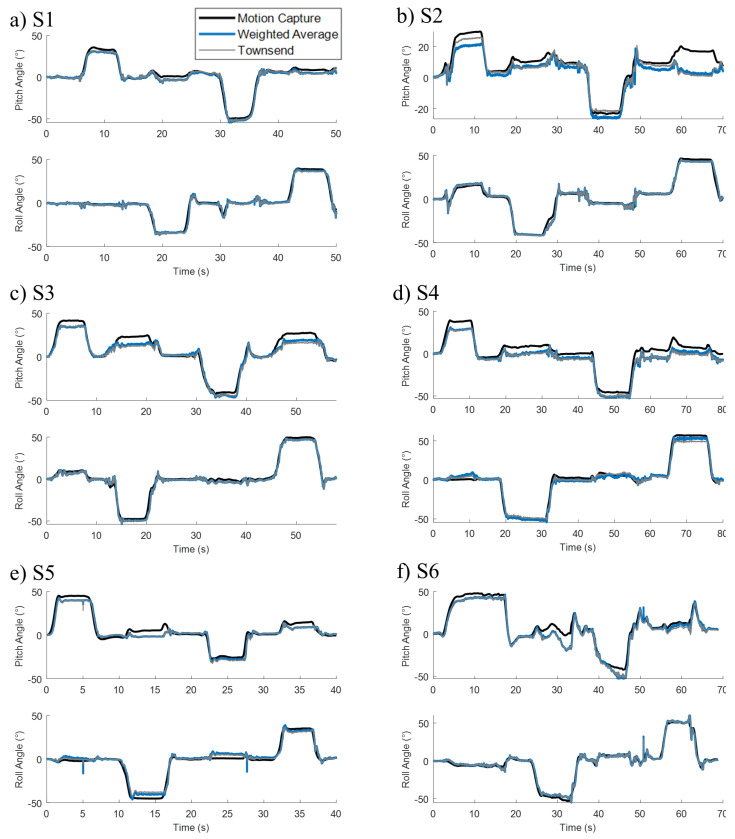
Pitch and roll angles from all subjects S1 (**a**), S2 (**b**), S3 (**c**), S4 (**d**), S5 (**e**), and S6 (**f**) determined through sensor fusion optimized with motion capture.

All subjects were able to reach the desired angles with the help of the experimenter or their upper extremities. No fatigue was observed during the movements. All of the subjects had some residual upper extremity function to aid in these movements. The average flexion, extension, right bending, and left bending angles were 37.8 ± 7.1°, −38.9 ± 10.9°, −45.3 ± 6.4°, and 45.5 ± 8.3°, respectively. Figure 5 shows the fused signals over a movement trial for all subjects. Both the weighted average and the Townsend algorithms tracked the target gold standard derived from the motion capture. An offset was present during the dwell stages of the movements. This occurred to the greatest degree during the flexion movements of S2, S3, and S4 with the maximum offset hovering around 10°.

The correlation coefficients (r) and root mean squared errors (RMSE) for the motion capture calibration are shown in Table 2. The r values for the weighted average equation ranged from 0.96 to 0.99 with the average being 0.98 ± 0.014 and 0.99 ± 0.004 for the pitch and roll directions, respectively. Those for the Townsend equation ranged from 0.94 to 0.99 with the average being 0.97 ± 0.019 and 0.99 ± 0.003 for the pitch and roll directions, respectively. The RMSE values for weighted average equation ranged from 2.12 to 5.1° with an average value of 4.01 ± 0.78° and 2.96 ± 0.78° for the pitch and roll directions, respectively. Those for Townsend equation ranged from 2.16 to 5.12° with an average value of 4.26 ± 0.86° and 3.03 ± 0.82° for the pitch and roll directions, respectively.

**Figure 6:**
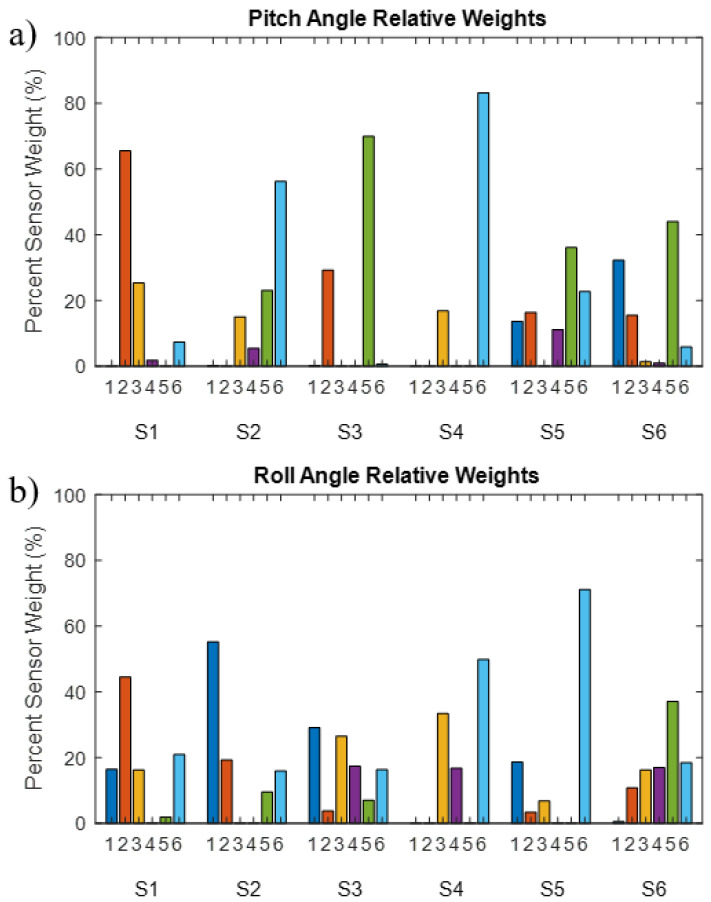
Sensor weights for determining the pitch (**a**) and roll (**b**) angles from the weighted average equation. Values are separated by subject number and sensor number. Locations of the sensors are as followed: 1. right chest; 2. left chest; 3. right front abdomen; 4. left front abdomen; 5. right back abdomen; and 6. left back abdomen. These weights resulted from the motion capture calibration process.

To determine which sensors were prioritized by the calibration process we plotted the relative weights of each sensor for each subject in Figure 6. The algorithm incorporated information from between two to five sensors. No obvious trends appeared in these data.

### 3.2. Clinical Calibration

**Figure 7:**
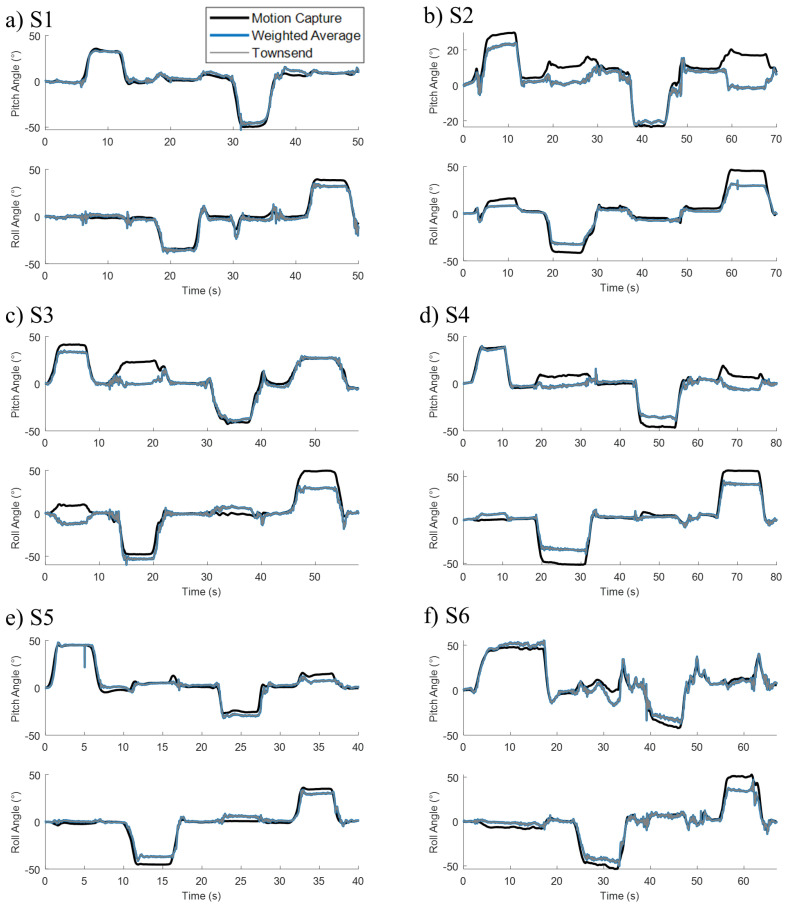
Pitch and roll angles from all subjects S1 (**a**), S2 (**b**), S3 (**c**), S4 (**d**), S5 (**e**), and S6 (**f**) determined through sensor fusion optimized with motion capture.

Figure 7 shows the fused signals over a movement trial for all subjects. Both the weighted average and the Townsend algorithms track the target gold standard derived from the motion capture. The clinical calibration method was still able to track trunk movement, however with greater errors especially evident in the dwell stages of movement.

The correlation coefficient (r) and root mean squared error (RMSE) for the clinical lab calibration are shown in Table 3. The r values for the weighted average equation ranged from 0.85 to 0.98, with the average being 0.93 ± 0.045 and 0.97 ± 0.024 for the pitch and roll directions, respectively. Those for the Townsend equation ranged from 0.85 to 0.98 with the average being 0.93 ± 0.045 and 0.97 ± 0.024 for the pitch and roll directions, respectively. The RMSE values for weighted average equation ranged from 3.63 to 9.57° with an average value of 7.14 ± 1.62° and 6.86 ± 2.14° for the pitch and roll directions, respectively. Those for Townsend equation ranged from 3.63 to 9.57° with an average value of 7.15 ± 1.62° and 6.86 ± 2.14° for the pitch and roll directions, respectively.

## 4. Discussion

An accurate measure of trunk orientation is a necessary component for robust design of feedback control systems for trunk stabilization with FNS after SCI. Current feedback control systems rely on a signal obtained from a single accelerometer sensor placed externally on the trunk. This process requires the user to don and doff the sensor, which could result in inconsistent placement from day to day. The implanted accelerometers from the NNP are capable of providing a feedback signal, however the location and orientation of these sensors are arbitrary as dictated by surgical constraints. In this paper, we have developed a method of fusing signals from multiple sensors with unknown orientations and locations into a single measure of trunk posture. Although the NNP is the inspiration for this study, the methods developed here would be applicable to applications determining trunk orientation from multiple sensors (implanted or surface mounted) without prior knowledge of their orientation relative to the body.

The motion capture calibration method shown here results in high correlation coefficients above 0.97 and RMSE values below 5°. These results are similar to other studies that measure trunk orientation which found errors ranging from 1 to 5° and correlation coefficients from 0.74 to 0.95 (Table 4) [30,31,32,33,34,35]. Two methods for fusing the sensor signals were explored: weighted average and Townsend equation. There were only minor differences, of the order of a tenth of a degree for the RMSE, between the two. As a result, either of these equations would be viable as a sensor fusion algorithm. We recommend the weighted average as implementation was simpler and the weights provide a more intuitive understanding of the relative importance of each sensor. To fuse six sensors, the weighted average equation has 17 computational operations compared to the Townsend equation with 425 operations, which could impact real time implementation. The Townsend equation was originally designed to combine different measures of heart rate by weighting them based on their residual error [39]. In this study, only the equation structure was used and the constants were determined through optimization. Future work could explore methods for predicting a sensor’s error through characterization of the noise or a Kalman filter. The Townsend equation could then be employed with constants based on predicted error at each time point.

The fidelity of the clinical calibration method was less than the motion capture calibration. With errors of 7° exceeding 20% of the targeted 30° leaning postures. We expected greater errors, as optimizing the algorithm directly to the target measurement will always yield better results when compared to optimizing based on a proxy measurement. Nonetheless, the clinical calibration indicates that this procedure can be performed with minimal materials, only requiring a hand-held goniometer to measure trunk orientation, adequately trained staff or caregivers, and a computer to record the signals and perform the optimizations. The process is simple enough that, with proper training, it could be performed at home by the NNP users or their caregivers and all computations carried out with the smartphone application that will be provided to all recipients [26]. Possibly, the motion capture calibration could be performed initially and if there is any signal degradation, the home calibration could be performed to fine-tune the initially derived constants.

During calibration, we determined each sensor’s pitch and roll angle by measuring the inclination of the gravity vector. This method assumes the accelerations from any movement are small. Figure 5c shows that at times where the subject is initiating movement and accelerating to a new position, the resulting sensor signals show greater fluctuations compared to dwell postures. Luinge et al. [34] addressed the body accelerations with a Kalman filter designed to differentiate body accelerations from gravity, which reduced RMSE of trunk orientation from 3 to 2°. We assumed trunk accelerations will be relatively low during seated activities, such as leaning. However, for high acceleration tasks, inclination Equations 6 and 7 could be replaced with a Kalman filter. The remaining calibration process could remain unchanged.

We found RMSE of below 5 and 8° for the motion capture and clinical calibration methods respectively. Crago et al. suggested that resolutions of 5, 3, and 5° would be acceptable for control of the hip, knee, and ankle during walking [40]. Feedback control of trunk movements likely requires less fidelity than walking as a seated posture is inherently more stable than standing. Assuming a trunk length of 50 cm (S1 to Posterior Superior Iliac Spine [41]) and a leaning target of 30° pitch, the C7 vertebrae would be extended 25 cm. If, for example, there was an error of −10° over double the error observed in this study, then the C7 vertebrae would be extended 17 cm. In this case, the workspace of the user would still be significantly extended, and the individual would be able to compensate with their upper extremities. If, instead, the offset was positive, the individual would be dwelling farther away from upright thus extending their workspace at the expense of requiring greater muscle forces. The errors observed here tended to undershoot the orientation of the trunk (Figure 5). Additionally, a controller could be envisioned that only activates once the user assumes their desired posture. This would account for possible inaccuracies and allow the user to set the posture based on the task at hand. Future work should incorporate the calibration method in feedback control of seated balance. Previous trunk-based feedback control systems have relied on a single accelerometer placed at C7 [18,19]. The algorithm shown here has the same outputs as the single accelerometer from previous studies. Therefore, we predict that multiple accelerometers with calibration could directly replace the single accelerometer without detriment to the controller.

### Limitations and Future Work

A limitation of this sensor fusion method is the inability to measure axial rotation of the trunk. Our quasi-static analysis determined trunk pitch and roll based on the inclination of the gravity vector. The accelerometers are blind to axial rotations because there is no change in accelerometer signal while the accelerometer is rotated about the gravity vector. The majority of IMUs are also equipped with a gyroscope to address this deficiency by measuring angular velocity. Currently, the NNP only contains accelerometers and adding gyroscopes to future incarnations of the system would allow for measurement of axial rotation. However, the addition would likely have a minimal effect on the accuracy of trunk pitch and roll angles. Luige et al. was able to determine trunk orientation from only a three-axis accelerometer with 2° RMSE [34]. A later addition of a gyroscope to their method did not result in a substantial change of accuracy [33]. Another advantage of using only accelerometers is the potential reduction in power demand. In a full IMU the accelerometer requires 5% of the power consumption, gyroscope 80%, and magnetometer 7% [20]. Even running six accelerometers simultaneously would result in less power consumption compared to a single gyroscope.

The calibration method here was only tested with individuals with SCI, as a paralyzed trunk represents a more difficult test scenario and the most relevant. There is no reason to believe this method would not work in the able-bodied population as well. However, proof in individuals with SCI is all that is required to move forward with implanted sensors for trunk control. Additionally, our sample size was small (N = 6) and skewed older. This study represents an initial proof of concept, and the calibration process will be applied to future individuals who receive a trunk-based neuroprosthesis, thus increasing the sample size over time. Increasing the sample size will help confirm the generalizability of this method. However, we only need to generalize to individuals that would benefit from improved seated balance, which we specifically targeted in this study.

The reliability of these measurements over the course of hours or multiple days was not determined in this study. Instead, the focus was on developing the fusing process. IMU drift occurs from integrating gyroscope data that results in a random errors as the integration amplifies noise [22,42]. Due to the lack of gyroscopes in this analysis our method may be less sensitive to the sources of random drift. Accelerometers are also known to be temperature dependent [43]. We do not expect this to be an issue with an implanted sensor, as there will be little temperature variation inside the body. However, these assumptions and the sensor fusion process need to be tested in a recipient of the NNP system. The quality of trunk orientation measurements will need to be assessed over the course of multiple days and months to see how often the system needs re-calibration.

## 5. Conclusions

We have shown a method of determining trunk posture from multiple externally placed accelerometers with random orientation and location. The procedure is developed with considerations for future clinical implementation that contains multiple implanted accelerometers placed in unknown orientations due to surgical constraints. The procedure can be calibrated either with a motion capture system to obtain measures of trunk pitch and roll with under 5° RMSE and correlation coefficients above 0.97 or using tools typically available in the clinical or home settings to obtain calibrations with RMSE under 8° and correlation coefficients above 0.93. Once the process is implemented with a subject with implanted sensors, the feedback signal can be employed in trunk control systems to resist internal or external perturbations and facilitate leaning movements, thus eventually providing more options for people with SCI using FNS for trunk stability.

## Figures and Tables

**Table 1:** Clinical characteristics of the study participants.

Subject	Age (y)	Gender	Height (cm)	Weight (kg)	Injury Level	AIS * Grade	Time Post Injury (y)
S1	50	F	168	58.5	C7	B	24
S2	69	M	168	77.1	T5	D	5
S3	59	F	173	84.9	C4–C7	C	4
S4	46	F	173	84.9	T4	A	10
S5	62	M	191	93.8	T11	B	12
S6	31	M	188	66.2	C5	C	10

* American Spinal Injury Association Impairment Score (AIS).

**Table 2:** Correlation coefficients (r) and root mean squared error (RMSE) from the motion capture calibration process. Individual results from each subject is shown along with the bolded summary statistics.

Subject	Correlation Coefficient (r)				RMSE (°)			
	Pitch		Roll		Pitch		Roll	
	Weighted	Townsend	Weighted	Townsend	Weighted	Townsend	Weighted	Townsend
S1	0.991	0.991	0.994	0.993	2.69	2.73	2.12	2.16
S2	0.958	0.940	0.993	0.993	4.13	4.96	2.46	2.50
S3	0.983	0.981	0.996	0.996	4.26	4.52	2.25	2.25
S4	0.984	0.983	0.993	0.992	3.81	3.99	3.44	3.88
S5	0.959	0.959	0.983	0.986	5.10	5.12	3.98	3.91
S6	0.984	0.983	0.990	0.991	4.04	4.26	3.52	3.50
**Average**	**0.976**	**0.973**	**0.991**	**0.992**	**4.01**	**4.26**	**2.96**	**3.03**
**STD**	**0.014**	**0.019**	**0.004**	**0.003**	**0.78**	**0.86**	**0.78**	**0.82**

**Table 3:** Correlation coefficients (r) and root mean squared error (RMSE) from the clinical calibration process. Individual results from each subject is shown along with the bolded summary statistics.

Subject	Correlation Coefficient (r)				RMSE (°)			
	Pitch		Roll		Pitch		Roll	
	Weighted	Townsend	Weighted	Townsend	Weighted	Townsend	Weighted	Townsend
S1	0.972	0.972	0.984	0.984	4.83	4.83	3.63	3.63
S2	0.847	0.847	0.968	0.968	8.25	8.25	7.26	7.26
S3	0.931	0.931	0.925	0.925	8.89	8.89	9.57	9.57
S4	0.931	0.938	0.995	0.995	8.02	8.06	8.11	8.11
S5	0.959	0.959	0.979	0.979	5.52	5.53	5.12	5.12
S6	0.957	0.957	0.981	0.981	7.35	7.35	7.49	7.49
**Average**	**0.934**	**0.934**	**0.972**	**0.972**	**7.14**	**7.15**	**6.86**	**6.86**
**STD**	**0.045**	**0.045**	**0.024**	**0.024**	**1.62**	**1.62**	**2.14**	**2.14**

**Table 4:** Comparison of previous work with the results from our study (first two rows).

Source	Activity	Population	Sensor	r	RMSE (°)
Motion Capture Calibration	Leaning	Individuals with SCI	Six 3-axis accelerometers	0.97	5
Clinical Calibration	Leaning	Individuals with SCI	Six 3-axis accelerometers	0.93	7
Mazza et al. [31]	Walking	Able-bodied	9-axis IMU	0.91	1
Punchihewa et al. [32]	Baseball hitting	Able-bodied	Two 9-axis IMU	0.95	5
Grimpampi et al. [30]	Walking	Individuals with hemiplegia or Parkinson’s	3-axis gyroscope	0.74	1.3 plus a 2 offset
Luinge et al. [33]	Lifting crates	Able-bodied	6-axis IMU	N/A	3
Luinge et al. [34]	Lifting crates	Able-bodied	3-axis accelerometer	N/A	2
Brouwer et al. [35]	Dynamic sport motions	Able-bodied	Two 9-axis IMU	0.85	5

## Data Availability

The data presented in this study are available on request from the corresponding author. The data are not publicly available due to source data participant consent limitations.

## Abbreviations

The following abbreviations are used in this manuscript:

FNS	Functional Neuromuscular Stimulation
SCI	Spinal Cord Injury
AIS	American Spinal Injury Association Impairment Score
NNP	Networked Neuroprosthesis
IMU	Inertial Measurement Unit
RMSE	Root Mean Squared Error
r	Correlation Coefficient
